# Fabrication of Al-Ni Alloys for Fast Hydrogen Production from Hydrolysis in Alkaline Water

**DOI:** 10.3390/ma16237425

**Published:** 2023-11-29

**Authors:** JaeYoung Kwon, KwangSup Eom, MinJoong Kim, Ihsan Toor, SeKwon Oh, HyukSang Kwon

**Affiliations:** 1 Department of Materials Science and Engineering, Korea Advanced Institute of Science and Technology, Daejeon 34051, Republic of Korea; jykwon@kaist.ac.kr (J.K.); ihsan@kaist.ac.kr (I.T.); 2 Department of Materials Science and Engineering, Gwangju Institute of Science and Technology, Gwangju 61005, Republic of Korea; keom@gist.ac.kr; 3 Hydrogen Research Department, Korea Institute of Energy Research, 152 Gajeong-ro, Yuseong-gu, Daejeon 34129, Republic of Korea; mj.kim@kier.re.kr; 4 Research Institute of Advanced Manufacturing Technology, Surface R&D Group, Korea Institute of Industrial Technology, Incheon 21999, Republic of Korea

**Keywords:** Al-Ni alloys, hydrogen generation, galvanic corrosion, intergranular corrosion

## Abstract

Hydrogen generation through the hydrolysis of aluminum alloys has attracted significant attention because it generates hydrogen directly from alkaline water without the need for hydrogen storage technology. The hydrogen generation rate from the hydrolysis of aluminum in alkaline water is linearly proportional to its corrosion rate. To accelerate the corrosion rate of the aluminum alloy, we designed Al-Ni alloys by continuously precipitating an electrochemically noble Al_3_Ni phase along the grain boundaries. The Al-0.5~1 wt.% Ni alloys showed an excellent hydrogen generation rate of 16.6 mL/${\mathrm{cm}}^{2}\cdot \min$, which is about 6.4 times faster than that of pure Al (2.58 mL/${\mathrm{cm}}^{2}\cdot \min$). This excellent performance was achieved through the synergistic effects of galvanic and intergranular corrosion on the hydrolysis of Al. By raising the solution temperature to 50 °C, the optimal rate of hydrogen generation of Al-1 wt.% Ni in 10 wt.% NaOH solutions at 30 °C can be further increased to 54.5 mL/${\mathrm{cm}}^{2}\cdot \min$.

## 1. Introduction

Hydrogen is generating significant interest as a prospective energy source to replace existing fossil fuels due to its numerous benefits, including its environmentally friendly nature, high energy density, and zero emissions [1,2]. However, most hydrogen production methods currently rely on chemical processes involving fossil fuels such as coal or natural gas reforming, leading to issues related to CO_2_ emissions [3]. Therefore, developing environmentally friendly hydrogen production methods is crucial for its use as clean energy. Hydrolysis of chemical hydride [4,5,6,7,8,9,10,11], active metals [12,13,14,15,16,17,18,19,20,21], and electrolysis [22,23,24,25,26,27,28,29] methods have been studied as environmentally clean hydrogen production methods. Among them, the on-board hydrogen production via the hydrolysis of electrochemically active metals, like Al [14,17,18,19,20,21] and Mg [12,13], has gained significant attention as it eliminates the necessity for hydrogen storage. Among the active metals, Al is a very suitable element for the production of hydrogen from its hydrolysis, due primarily to the fact that it is light, cheap (1~3 USD/kg), abundant, and electrochemically very active (E°_Al3+/Al_ = −1.8 V_SHE_) [17,18,19,20,21]. The chemical reaction involving the hydrolysis of Al, as expressed in Reaction (1), indeed takes place in an alkaline solution through the consecutive reactions outlined in Reactions (2) and (3). Approximately 1 kg of Al produces around 0.110 kg of H_2_, equivalent to 1340 L of H_2_ gas (at 1 atm, 298 K) [17,18,19,20,21] through Reaction (1) that occurs via consecutive reactions of Reactions (2) and (3).

Al + 3 H_2_O → Al(OH)_3_ + 3/2 H_2_ (1)

Al + 3 H_2_O + NaOH → NaAl(OH)_4_ + 3/2 H_2_ (2)

NaAl(OH)_4_ → NaOH + Al(OH)_3_ (3)

Most of the previous research on the hydrolysis of Al has been conducted using powders of Al and its alloys due primarily to their fast hydrogen production kinetics [15,16,18,19]. However, they are dangerous owing to their high reactivity with moisture. They are also expensive for commercialization [30]. Bulk aluminum or aluminum alloys like sheets and plates are relatively cost-effective and safe. Nevertheless, they display inadequate hydrogen generation kinetics, making them unsuitable for commercialization. [31]. Therefore, it would be a challenge to develop safe and inexpensive bulk-type Al alloys with fast hydrogen generation kinetics. The hydrogen production rate is fundamentally dependent on the corrosion or oxidation rate of aluminum in alkaline water as indicated in Reaction (1). Based on this fundamental concept, we previously developed an Al-1 wt.% Fe alloy. In this alloy, an electrochemically noble Al_3_Fe phase continuously precipitates along the grain boundaries. Consequently, the corrosion rate of aluminum experiences a substantial increase due to the synergistic effects of galvanic and intergranular corrosion between these noble precipitates and the matrix phase [20]. It is anticipated that the cooperative influence of galvanic and intergranular corrosion on the rate of hydrogen generation through the hydrolysis of aluminum will be enhanced by the formation of electrochemically nobler precipitates along the grain boundaries, surpassing the Al_3_Fe present in the Al-1 wt.% Fe alloy. The Al_3_Ni precipitated in Al-Ni alloy is an electrochemically more noble phase than the Al_3_Fe formed in Al-Fe alloy [32], and hence the hydrogen generation rate in an optimally designed Al-Ni alloy would be much faster than that in Al-1 wt.% Fe alloy with a lower content of Ni. The objective of this study is to develop an optimum Al-Ni alloy in which an electrochemically noble phase like Al_3_Ni forms along the grain boundaries. This aims to significantly enhance the hydrogen generation kinetics from the hydrolysis of the Al alloy through the synergistic effects of galvanic and intergranular corrosion.

## 2. Experimental

The Al-xNi alloys (x = 0~1.5) were prepared by melting pure Al (99.9%) and Al-Ni master alloy at 1200 °C. The Al-Ni master alloy used for the fabrication of the alloys contains 40 wt.% of Ni whose phase is expected to be Al_3_Ni based on the Al-Ni binary phase diagram. The content of Ni in Al-xNi alloys (0, 0.5, 1, 1.5 wt.%) was adjusted by varying the weight of the Al-Ni master alloy, and hence each alloy is termed Al-0.5Ni, Al-1Ni, and Al-1.5Ni, depending on the Ni content in this work. Then, the Al alloys were air-cooled in a stainless-steel mold to precipitate the intermetallic compound (Al_3_Ni) along the grain boundary. The microstructure of the alloys was examined by etching them chemically in 10 wt.% NaOH solution for 10 s at room temperature. All samples were fabricated with dimensions of 5 × 5 × 5 cubic millimeters. The surface area exposed remained constant at 1.5 square centimeters. Surface morphology and compositional analysis of the Al-Ni alloys were performed using scanning electron microscopy (SEM) and energy-dispersive spectroscopy (EDS). The microstructure of these alloys was confirmed via X-ray diffraction (XRD) and transmission electron microscopy (TEM). Polarization curves of the pure Al and Al_3_NI alloys, respectively, were measured in 10 wt.% NaOH solution at 25 °C with a scan rate of 1 mV/s. The electrochemical cell equipped with a platinum counter electrode, a saturated calomel reference electrode (SCE), and a working electrode (exposed area: 0.1256 cm^2^) were used for the polarization tests. To investigate the galvanic corrosion behavior of aluminum when coupled to Al_3_Ni, polarization tests were conducted in a 0.1 M NaOH solution at 30 °C. The galvanic corrosion rate of the aluminum, electrically connected to Al_3_Ni, was measured using a zero-resistance ammeter (ZRA) in the same 0.1 M NaOH solution at 30 °C. The exposed surface areas of aluminum and Al_3_Ni were maintained at a 1:1 ratio. Hydrogen generation from the hydrolysis of Al-Ni alloys was tested in a 50 mL solution containing 10% NaOH at a temperature of 30 °C. The quantity of hydrogen gas produced from the hydrolysis of the Al-Ni alloys was measured using a mass flow meter (MFM). Furthermore, the study examined how changes in NaOH concentration and solution temperature affected the rate of hydrogen generation from the Al-Ni alloy.

## 3. Results and Discussion

**Figure 1:**
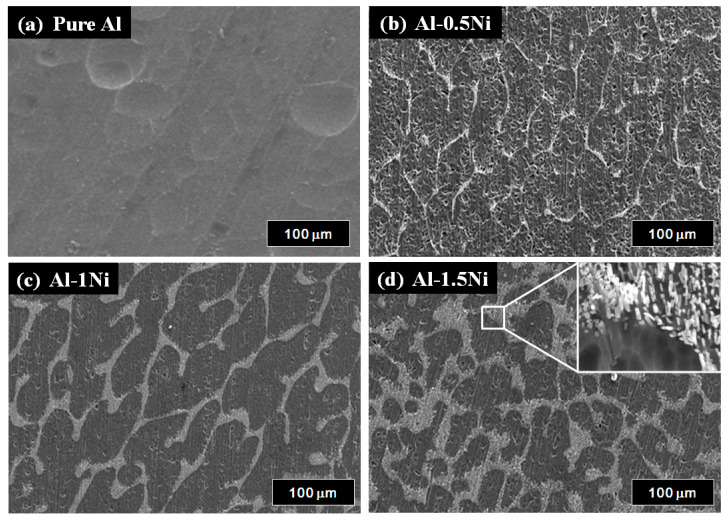
SEM images showing the surface morphologies of Al-xNi alloy (x = 0, 0.5, 1, 1.5 wt.%): (**a**) pure Al, (**b**) Al-0.5Ni, (**c**) Al-1.5 Ni, (**d**) Al-2.0 Ni with inset showing magnified image of precipitates at the grain boundary areas. All of the Al-Ni alloys were slightly etched in 10 wt.% NaOH solution for 10 s before imaging.

**Figure 2:**
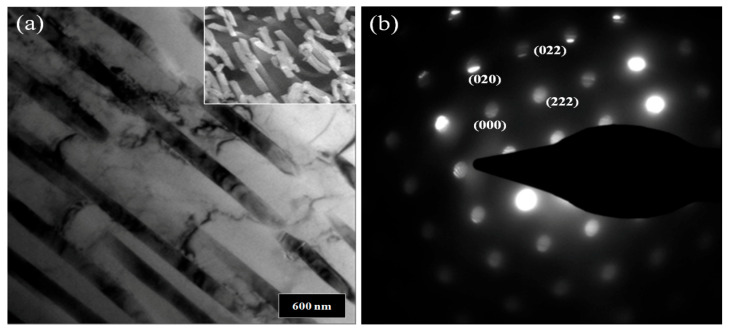
(**a**) TEM bright field images on the precipitates of Al-1.5Ni with an inset showing their SEM image. (**b**) Diffraction pattern of an Al-1.5Ni precipitate, which confirms that the precipitates formed at the grain boundaries is Al_3_Ni.

**Figure 3:**
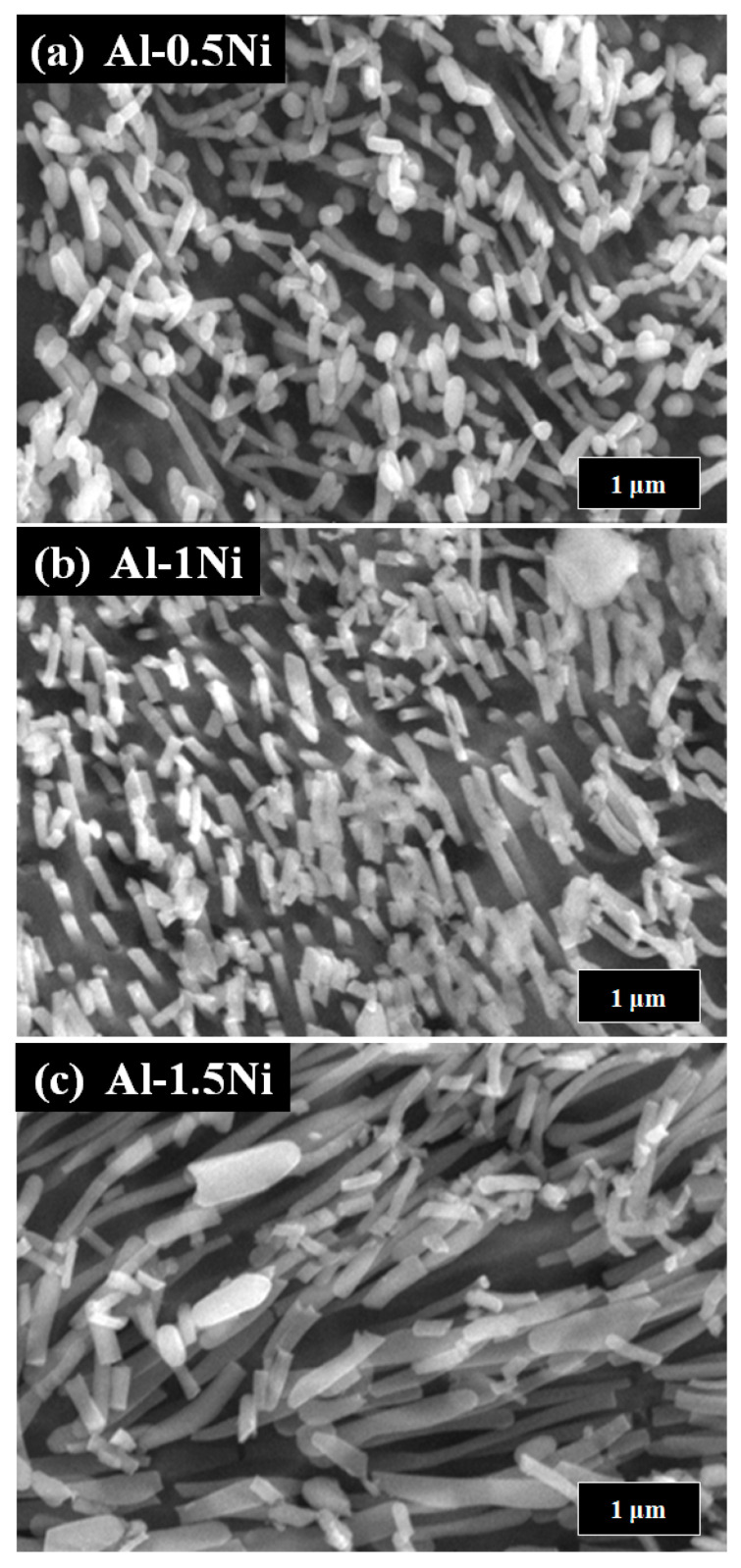
SEM images of precipitates formed at the vicinity of grain boundaries of Al-x Ni alloys (x = 0.5~1.5 wt.%): (**a**) Al-0.5Ni, (**b**) Al-1Ni, and (**c**) Al-1.5Ni.

**Figure 4:**
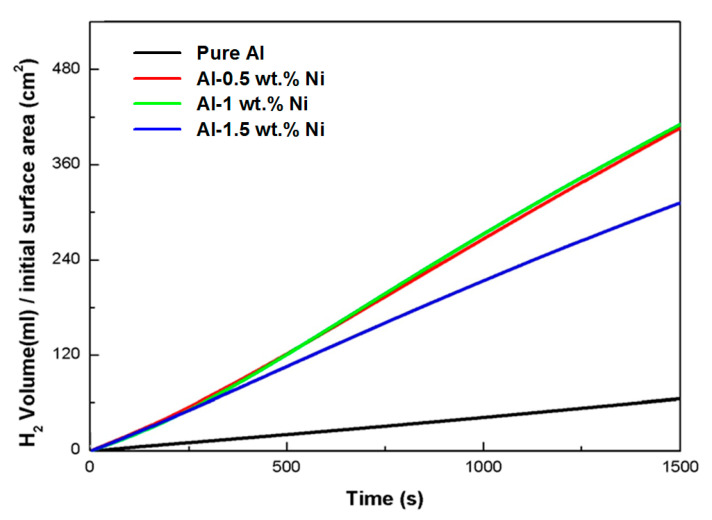
Effects of Ni content on the hydrolysis reaction of pure Al (black), Al-0.5Ni (red), Al-1Ni (green), and Al-1.5Ni (blue) in 10 wt.% NaOH solution at 30 °C.

**Figure 5:**
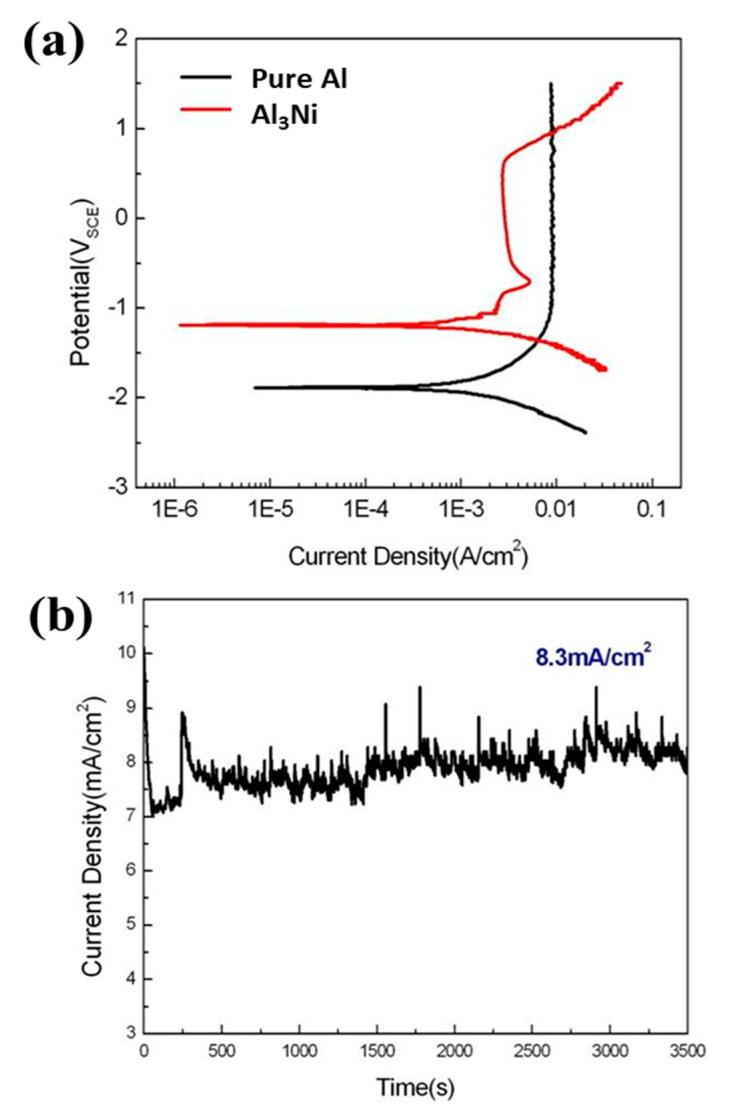
(**a**) Polarization behaviors of pure Al and Al_3_Ni, (**b**) galvanic current density of Al electrically coupled to Al_3_Ni in 0.1 M NaOH solution at 30 °C.

**Figure 6:**
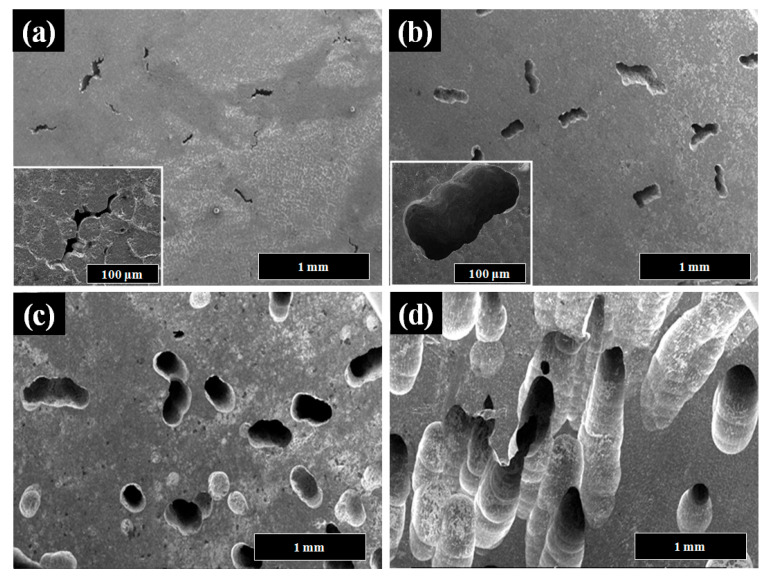
SEM images on the pits formed on the Al-1Ni after (**a**) 1 min, (**b**) 5 min, (**c**) 30 min, and (**d**) 60 min of hydrolysis, respectively, in 10 wt.% NaOH solution at 30 °C.

**Figure 7:**
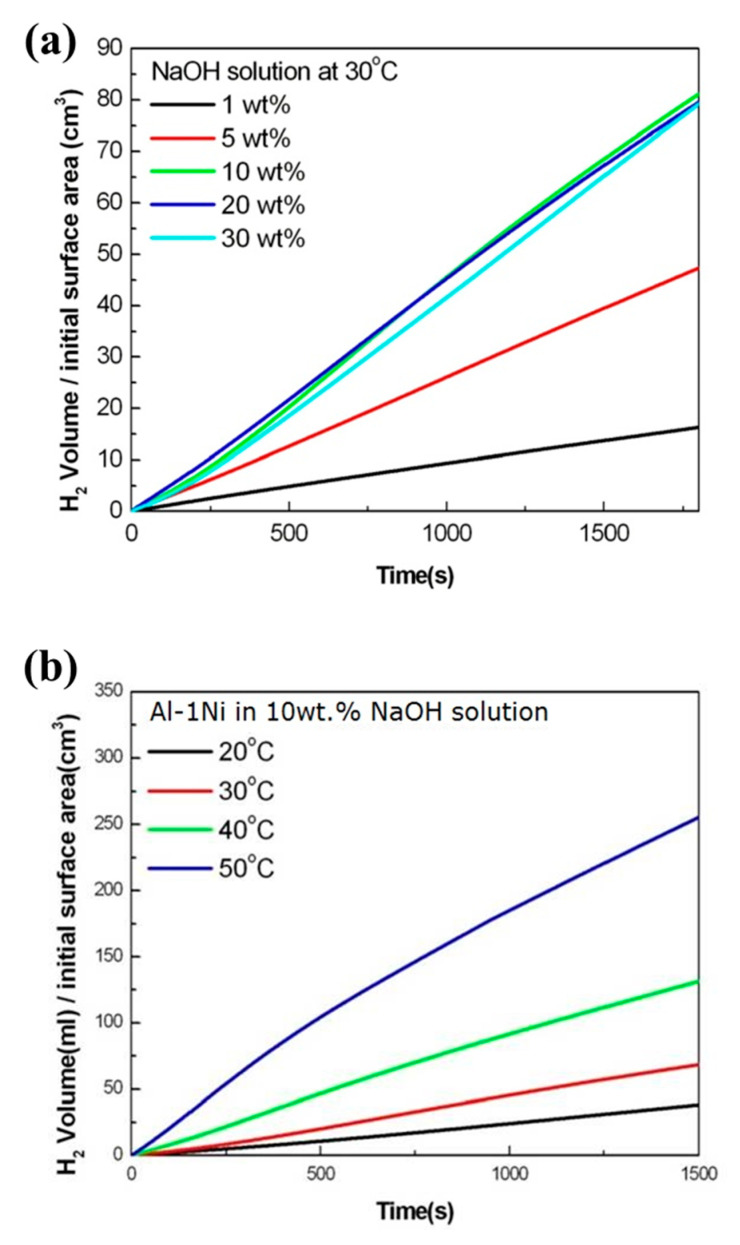
(**a**) Effects of NaOH concentration on the hydrolysis of Al-1Ni; 1 wt.% (black), 5 wt.% (red), 10 wt.% (green), 20 wt.% (blue), and 30 wt.% (light blue) at 30 °C. (**b**) Effects of solution temperature on the hydrolysis of Al-1Ni alloy in 10 wt.% NaOH solution with the temperature at 20 °C (black), 30 °C (red), 40 °C (green), and 50 °C (blue).

Figure 1 shows the surface morphologies of pure Al and Al-Ni alloys after being polished and etched for 1 min in 10 wt.% NaOH solution. Figure 1a shows a smooth morphology of pure Al. With Ni content in Al alloys, the precipitates were observed on the surface of Al-Ni alloys as shown in Figure 1b–d. From the EDS analysis (Table 1), the bright region along the grain boundaries indicates Al-Ni precipitates, or an intermetallic compound between Al and Ni based on the phase diagram of Al-Ni, whereas the dark region is pure Al matrix. As the Ni contents are increased from 0.5 to 1.5 wt.%, the bright area where the precipitates form is increased. For Al-0.5Ni and Al-1Ni alloys, the precipitates formed exclusively along grain boundaries, whereas for the Al-1.5Ni alloy, they formed in the interior of grains as well as at grain boundaries. In the inset of Figure 1d, the precipitates formed at the grain boundaries of Al-1.5Ni look like needles with their length ranging from 1.5 to 2 μm. The precipitates were further examined using TEM, and the diffraction pattern of a precipitate shown in Figure 2b revealed that its phase is Al_3_Ni or the same phase as that of the starting master alloy (Al-40 wt.% Ni). Figure 3a–c reveal the magnified SEM images of the precipitates formed in the vicinity of the grain boundaries of Al-0.5Ni, Al-1Ni, and Al-1.5Ni. The images show that as the Ni content increases, the shape and the size of the precipitates change. When the Ni content is 0.5 wt.%, both elliptical and needle-shaped precipitates were formed over the grain boundaries with their length ranging from 0.3 to 0.4 μm, as shown in Figure 3a. As Ni content increases up to 1.5 wt.% Ni, the length of precipitates with a needle-like shape increases up to 1~2 μm, as shown in Figure 3c. Figure 4 shows the results of hydrogen generation tests of pure Al and the designed Al-Ni alloys from the hydrolysis of the alloys in 10 wt.% NaOH solution at 30 °C. The hydrogen generation, defined as the hydrogen volume normalized by the initial surface area of the bulk Al-Ni alloy, was recorded with time after having the alloys react with the NaOH solution for 750 s to obtain stabilized hydrolysis reaction data. Based on the data, the hydrogen generation rate for each alloy was calculated and presented in Table 2. With the Ni content increased from 0 (pure Al) to 1 wt.% in the Al-Ni alloys, the hydrogen generation kinetics significantly promoted from 2.58 mL/${\mathrm{cm}}^{2}\cdot \min$ to 16.6 mL/${\mathrm{cm}}^{2}\cdot \min$ that is ~6.4 times faster than that of pure Al, as shown in Table 2. However, when the Ni content exceeds 1 wt.% and reaches 1.5 wt.%, the hydrogen generation rate declined to 12.54 mL/${\mathrm{cm}}^{2}\cdot \min$, which is 4.9 times faster than that of pure Al. Therefore, it became clear that 0.5~1 wt.% Ni is the optimum level for the maximum hydrogen production among the examined alloys. The notable improvement in the hydrogen reaction rate of Al-Ni alloys is closely associated with the presence of Al_3_Ni precipitates that form along the grain boundaries. These precipitates can initiate galvanic corrosion of the aluminum due to the difference in corrosion potential between the Al matrix and the Al_3_Ni precipitate. Additionally, the continuous formation of Al_3_Ni precipitates along the grain boundaries may also lead to intergranular corrosion, particularly in regions proximate to the interface between the precipitate and the matrix. The acceleration in the corrosion rate of Al-Ni alloys increases the hydrogen generation kinetics of Al-Ni alloys. The hydrogen generation rate of Al-0.5~1Ni alloys, which is 6.4 times faster than that of pure Al, significantly surpasses the rates reported for Al-1 wt.% Fe [20] (3.8 times faster) and Al-5 wt.% Cu alloy (4.7 times faster), as previously documented [21]. Galvanic corrosion takes place when two metals with differing electrochemical properties are electrically connected in a corrosive solution [33,34,35]. In galvanic corrosion, the metal with a lower corrosion potential (E_corr_) functions as the anode and undergoes accelerated corrosion, while the metal with a higher corrosion potential serves as the cathode and is thus protected from corrosion. The rate of corrosion in galvanic corrosion scenarios is directly proportional to the difference in corrosion potential between the metals in the galvanic couple. In an alkaline solution, the corrosion potential of aluminum is significantly lower than that of other metals like Fe, Ni, Cu, Co, and their aluminides. Consequently, when electrically connected to these metals, aluminum acts as the anode, resulting in accelerated corrosion [20,21]. According to previous studies [20,21], particularly, Fe and Cu were found to be excellent for increasing the galvanic corrosion rate when coupled to Al. Electrochemical analysis of the Al and Al_3_Ni present in the Al-Ni alloys was carried out by measuring their individual E_corr_. Figure 5a shows the potentiodynamic polarization responses for Al and Al_3_Ni (Al-40 wt.% Ni) in 0.1 M NaOH solution at 30 °C. The test results listed in Table 3 show that the corrosion potential of Al and Al_3_Ni are −1.88 V_SCE_ and −1.19 V_SCE_, respectively. These results confirm that Al_3_Ni or the precipitates formed along the grain boundaries have an E_corr_ that is ~0.7 V_SCE_ noble to Al matrix, resulting in galvanic corrosion in the Al matrix when the Al-Ni alloys are exposed to alkaline water. The polarization curves in Figure 5a show that the corrosion current densities (i_corr_) of Al and Al_3_Ni are 1.94 mA/cm^2^ and 1.17 mA/cm^2^, respectively. For further analysis of the galvanic corrosion effect of the Al_3_Ni phase on the Al matrix, a galvanic corrosion test was carried out on the galvanic couple, consisting of Al and Al_3_Ni with the same exposed area using ZRA in 0.1 M NaOH solution at 30 °C. The galvanic current density plotted in Figure 5b shows that the corrosion rate of Al coupled to Al_3_Ni yields 8.3 mA/cm^2^, which is 4.3 times faster than that (1.94 mA/cm^2^) of pure Al. Hence, it became clear that the precipitates (Al_3_Ni) significantly enhance the corrosion rate of the Al phase in the alloys by the galvanic corrosion action. For a detailed evaluation of the corrosion behavior of Al-Ni alloy during its hydrolysis, the Al-1Ni alloy was immersed in 10 wt.% NaOH at 30 °C for 1 min, 5 min, 30 min, and 60 min, and then their surface morphologies were observed. Figure 6a shows the SEM image on the surface morphology of Al-1Ni when 1 min has elapsed from the beginning of hydrolysis. A few numbers of tiny pits were formed at the onset of hydrolysis, and the inset in Figure 6a or the magnified image of a pit clearly reveals that intergranular corrosion occurs at the initial stage of hydrolysis [33]. This is attributed to the fact that galvanic corrosion between the Al matrix and Al_3_Ni precipitates is accelerated in areas near the precipitates, primarily because of the shorter electrical circuit path with lower resistance. As more hydrolysis progresses with time, the ongoing intergranular corrosion causes the interior Al grains to fall off, and results in the formation of larger pits or pores. It is evident from Figure 6b that the number and size of pores increased in the Al-1Ni alloy after 5 min of hydrolysis. With the hydrolysis reaction going on, the reaction area of the alloy for the hydrolysis increases due to the enlarged pores and the ejected grains, and hence this, in turn, significantly enhances the hydrolysis rate. The SEM images in Figure 6 show that the diameter of the pores increases dramatically with hydrolysis, from 40~50 μm (1 min) to 300 μm (30 min, Figure 6c), and eventually the structure of the entire alloy becomes porous after 1 h hydrolysis, as demonstrated in Figure 6d. Therefore, from the electrochemical and surface analysis, the contribution of galvanic corrosion and intergranular corrosion to the increase in the hydrogen generation rate was calculated to be approximately 67% and 33%, respectively. The decrease in the hydrogen generation rate of Al-1.5 Ni alloy shown in Figure 5, compared with that of Al-1 Ni alloy, is due probably to the reduction in the contribution of the intergranular corrosion to the hydrolysis as discussed previously [19]. Besides the Ni content, the hydrogen generation rate of Al-Ni alloys is a function of NaOH concentration and solution temperature. Figure 7a shows the influences of NaOH concentration on the hydrogen generation rate of Al-1Ni at 30 °C. With the increase in NaOH concentration from 1 wt.% to 10 wt.%, the hydrogen generation rate was measured to increase significantly from 3.15 mL/${\mathrm{cm}}^{2}\cdot \min$ to 16.6 mL/${\mathrm{cm}}^{2}\cdot \min$. However, as the NaOH concentration was raised to 20 wt.% or 30 wt.%, the rate of hydrogen generation showed little change, much like the consistent levels observed in the 10 wt.% NaOH solution (as indicated in Table 4). These results showed that excess OH- ions do not contribute to the enhancement of the hydrolysis rate due probably to the increase in viscosity of the solution, and hence the optimum NaOH concentration for the fast hydrolysis of Al is 10 wt.%. Figure 7b and Table 5 show the effects of solution temperature on the hydrogen generation of Al-1Ni in 10 wt.% NaOH solution. Evidently, the hydrogen generation rate consistently increased with the solution temperature. For example, the hydrogen generation rate of Al-1Ni in 10 wt.% NaOH was measured to be 8.9 mL/${\mathrm{cm}}^{2}\cdot \min$ at 20 °C, and the rate increased to 53.5 mL/${\mathrm{cm}}^{2}\cdot \min$ by raising the solution temperature to 50 °C. The increase in the hydrogen generation rate is due to a reduction in the activation energy for the hydrolysis as the temperature rises. From the test results as shown in Figure 7b, it was measured that the hydrogen generation rate almost doubled as the temperature was increased by 10 °C.

## 4. Conclusions

The results of this work illustrate that low-cost and nonhazardous Al-Ni bulk alloy is able to serve as an excellent on-board hydrogen generator for fuel cell application via hydrolysis in alkaline water. The designed Al-0.5~1 wt.% Ni alloys composed of an Al matrix and Al_3_Ni precipitated along grain boundaries showed a significantly enhanced hydrolysis rate that is 6.4 times faster than that of pure Al, and also much higher than either that of Al-1 wt.% Fe alloy or that of Al-5Cu alloy. The increased rates of hydrogen generation in the Al-0.5~1 wt.% Ni alloys were accomplished through the synergistic effects of galvanic corrosion and intergranular corrosion between the Al matrix and Al_3_Ni precipitates. It was estimated that galvanic corrosion contributed to approximately 67% of the increase in the hydrogen generation rate, while intergranular corrosion accounted for the remaining 33% in the case of Al-1 wt.% Ni. With the hydrolysis of Al, the ongoing intergranular corrosion makes the interior Al grains fall off, thereby resulting in the formation of coarse pits or pores. The hydrogen generation rate of the Al-Ni alloys can be maximized by tuning Ni content, NaOH concentration, and solution temperature to optimum values. The designed Al-1Ni alloy yielded the best hydrogen generation rate of 16.6 mL/${\mathrm{cm}}^{2}\cdot \min$ in 10 wt.% NaOH solution at 30 °C, and this value can be further improved to 3.5 mL/${\mathrm{cm}}^{2}\cdot \min$ with the increase in temperature to 50 °C.

## Figures and Tables

**Table 1:** EDS analysis of a grain boundary area and interior region of a grain in Al-1.5Ni.

	Element	wt.%	at.%
**Bright region**	Al	89.39	93.78
	Ni	12.61	6.22
**Dark region**	Al	99.52	99.78
	Ni	0.48	0.22

**Table 2:** Hydrogen generation rate of Al-xNi alloys (x = 0~1.5 wt.%) in 10 wt.% NaOH solution at 30 °C.

Ni Content (wt.%)	Hydrogen Generation Rate (mL/cm^2^·min)
0	2.58
0.5	16.60
1	16.64
1.5	12.54

**Table 3:** Corrosion potential (E_corr_) and corrosion rate (i_corr_) of Al and Al_3_Ni, respectively, in 0.1 M NaOH solution at 30 °C.

	E_corr_ (V)	i_corr_ (mA/cm^2^)
**Al**	−1.88	1.94
**Al_3_Ni**	−1.19	1.17

**Table 4:** Hydrogen generation rate of Al-1Ni alloy with the change in NaOH concentration at 30 °C.

NaOH Concentration (wt.%)	Hydrogen Generation Rate (mL/cm^2^·min)
1	3.15
5	8.76
10	16.64
20	15.94
30	16.94

**Table 5:** Hydrogen generation rate of Al-1Ni alloy with the change in solution temperature in 10 wt.% NaOH solution.

Solution Temperature (°C)	Hydrogen Generation Rate (mL/cm^2^·min)
20	8.90
30	16.64
40	31.32
50	53.49

## Data Availability

Data is contained within the article.
